# Immune-metabolic positive feedback model in COPD: cross-mechanisms and potential intervention strategies

**DOI:** 10.3389/fcell.2026.1756033

**Published:** 2026-03-06

**Authors:** WenJing Chen, Shi Huang, Lijia He, Xin Zhou, RuiXiang Li, Guobing Wang

**Affiliations:** 1 Southwest Medical University, Luzhou, China; 2 The First Affiliated Hospital of Chongqing Medical University, Chongqing, China; 3 Yibin Traditional Chinese Medicine Hospital, Yibin, China

**Keywords:** chronic obstructive pulmonary disease, immunosenescence, inflammaging, metabolic reprogramming, mitochondrial dysfunction, NAD^+^–SIRT1 signaling, NLRP3 inflammasome

## Abstract

Chronic obstructive pulmonary disease (COPD) is a common chronic condition characterized by chronic bronchitis and/or emphysema with airflow obstruction, which can progress to cor pulmonale and respiratory failure. Associated with abnormal inflammatory responses to harmful gases and particulate matter, it carries high rates of disability and mortality, with a global prevalence among individuals aged 40 and older reaching 9%–10%. It is often regarded as a clinical and molecular model of accelerated lung aging. Age-related drift in immune function and metabolism plays a central part in this process, but how these changes are linked across different biological levels is still not fully clarified. Current work highlights mitochondrial injury and excessive reactive oxygen species as a central node that disrupts energy-sensing pathways, interferes with autophagy and epigenetic control, and weakens mitochondrial biogenesis, together fostering long-term glycolipid imbalance. At the same time, NF-κB–driven senescence-associated secretory activity and mitochondrial damage signals that engage the NLRP3 inflammasome form a reinforcing circuit that promotes macrophage dysfunction and exhaustion-like impairment of T and natural killer cells. These immune–metabolic disturbances stabilize low-grade chronic inflammation and metabolic instability, helping to explain persistent inflammatory sequelae, airway remodeling, and progressive decline in lung function. Building on these insights, we discuss a translational path centered on composite biomarker panels that integrate immune-exhaustion signatures, senescence mediators, NAD+–SIRT1 status, mitochondrial injury markers, and NLRP3 activity, and we consider low-intensity, multi-target therapeutic strategies designed to overcome the limitations of single-pathway treatments in COPD.

## 1 Introduction

Chronic obstructive pulmonary disease (COPD) is a highly disabling respiratory disorder that predominantly affects older individuals (Hoke et al., 2024). It is increasingly regarded not only as a clinical entity but also as a molecular model of accelerated lung aging (Glaab and Taube, 2011). Lung tissue from patients with COPD frequently displays key aging features, including telomere shortening, impaired mitochondrial respiration, disturbed nutrient-sensing pathways, expansion of senescent cell populations, and persistent low-grade inflammation, that together drive gradual loss of lung function and structural airway remodeling (Barnes, 2019).

Immune aging in COPD involves both innate and adaptive arms. Circulating and airway T cells commonly show exhaustion-like or senescent phenotypes (Li et al., 2022). NK-cell cytotoxicity is reduced, alveolar macrophages exhibit defective phagocytosis and autophagy (Barnes et al., 2022). Senescence-associated secretory phenotype (SASP) activity is increased (Roth-Walter et al., 2024; Jirru et al., 2020).

At the metabolic level, widespread mitochondrial damage and excessive production of mitochondrial reactive oxygen species (mtROS) are considered key drivers of COPD progression (Zinovkin et al., 2014). These alterations compromise oxidative phosphorylation and promote the release of damage-associated molecular patterns (DAMPs), which can activate NLRP3 signaling (Effah et al., 2021). Immune cells may shift from predominantly oxidative metabolism toward glycolysis, accompanied by disturbed lipid handling (Meng and Bozec, 2018). Hyperactivation of mTOR and reduced AMPK activity (Zhang et al., 2025), along with downregulated NAD^+^–SIRT1 signaling, further intensify oxidative stress, limit mitochondrial autophagy, and accelerate cellular senescence (Rivas et al., 2022).

**FIGURE 1:**
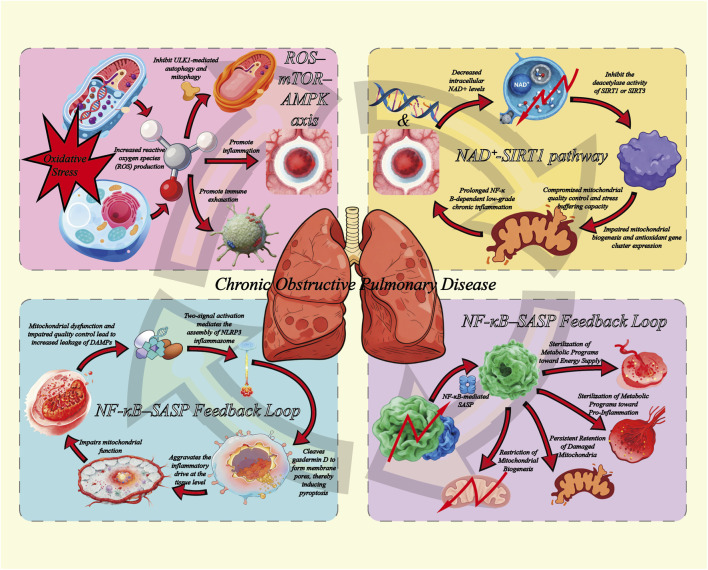
Immune–metabolic positive feedback loop model.

Despite substantial progress, many studies still examine immune aging and metabolic dysregulation in isolation. As a result, the mechanisms that integrate these processes and amplify them within the pulmonary microenvironment remain only partly defined. This systematic review therefore brings together key phenotypic and signaling features of immunosenescence and metabolic reprogramming in COPD. We synthesize evidence across the ROS–mTOR–AMPK axis, the NAD^+^–SIRT1 pathway, NF-κB–SASP–driven inflammatory reinforcement, and mitochondrial DAMP–NLRP3 activation (Fu et al., 2025). On this basis, we propose a testable immune–metabolic positive-feedback model (Figure 1) and discuss related biomarkers, multi-target treatment approaches, and implications for stratified COPD management and precision interventions that target aging biology.

## 2 Immunosenescence in COPD

### 2.1 Degeneration of the innate immune system

Innate immune decline in COPD is prominently reflected in senescent alveolar macrophages (Kojima et al., 2013). They show impaired microbial and apoptotic-cell clearance, defective autophagy/mitochondrial quality control (Roth-Walter et al., 2024), and heightened release of SASP mediators (e.g., IL-6, TNF-α, CXCL8) (Kubo et al., 2019), which are further aggravated by cigarette smoke (Kojima et al., 2013). Neutrophils exhibit excessive mtROS and NETosis, damaging the mucosa and increasing exacerbation risk (Trivedi et al., 2021). Natural killer cells also become dysregulated, with reduced systemic cytotoxicity but enhanced lung-resident cytotoxicity that can injure epithelium (Prieto et al., 2001). Together, these changes weaken first-line defense and destabilize tissue homeostasis, sustaining a chronic inflammatory milieu typical of immune aging in COPD (Shaikh et al., 2023).

### 2.2 Decline of adaptive immune function

Adaptive immunity shows overlapping features of immunosenescence and exhaustion. Senescent/terminally differentiated T cells commonly lose CD28 and upregulate CD57/KLRG1, whereas exhaustion is best defined as a state of persistent antigen/inflammatory exposure with sustained inhibitory receptors (e.g., PD-1, TIM-3), reduced proliferative capacity, and impaired cytokine or cytotoxic function (Fernandes, 2022). In COPD, both patterns may coexist in airway and circulating lymphocytes, and rigorous use of the term exhaustion should therefore rely on combined evidence from phenotype, transcriptome, and function (e.g., stimulation-induced cytokine production or killing assays), rather than marker expression alone (Ramos Jesus et al., 2024). CD4^+^ T cells often skew toward Th17 with functional Treg impairment, reflected by increased IL-17 and reduced IL-10, which contributes to infection susceptibility and tissue injury (Lourenço et al., 2021).

B cells also participate via ectopic tertiary lymphoid structures and strengthened CXCL13–CXCR5 signaling, indicating sustained antigenic stimulation and remodeling pressure (Xiong et al., 2020). Overall, terminal differentiation, exhaustion-like dysfunction, and Th17–Treg imbalance correlate with COPD severity, exacerbation risk, and structural lung damage, forming a key adaptive basis of COPD-associated immune aging.

### 2.3 Persistent inflammation and SASP reinforcement

NF-κB–dependent SASP production links innate and adaptive immune aging in COPD (Kumar et al., 2014). In smoke- or DAMP-exposed macrophages and epithelial cells, NF-κB not only sustains SASP cytokines (notably IL-1β/IL-6/IL-8) but also primes inflammasome components, increasing NLRP3 and pro–IL-1β expression (Shimada et al., 2012). Meanwhile, mitochondrial dysfunction and mtROS promote release of mitochondrial DAMPs (e.g., oxidized mtDNA, cardiolipin) (Shen, 2022), which provide the activation signal for NLRP3–caspase-1, driving IL-1β/IL-18 maturation and further NF-κB/SASP amplification (Shimada et al., 2012). These cytokines and ROS disrupt mTOR–AMPK balance and deplete NAD^+^, weakening SIRT1-dependent mitochondrial quality control and autophagy (Xie et al., 2020). The result is a reinforcing circuit in which SASP and DAMP–NLRP3 signaling reciprocally maintain inflammation and mitochondrial injury, promoting immune-cell dysfunction and metabolic drift (Wang et al., 2024).

To validate this proposed reinforcement loop in patients, studies should test whether SASP/NLRP3 activity and mitochondrial-injury markers co-localize in the same lung niches and track together over time, and whether targeted perturbations (e.g., restoring mitophagy, inhibiting NLRP3, or dampening NF-κB/SASP) break the loop and improve phagocytosis or lymphocyte function in models (Ning et al., 2022; Kotlyarov, 2025).

Overall, persistent ROS perturbs energy-sensing pathways by stimulating mTOR, inhibiting AMPK, and depleting NAD^+^, which weakens SIRT1-dependent anti-inflammatory signaling and mitochondrial quality control (Xie et al., 2020). These changes further reinforce SASP–NLRP3 amplification and favor a shift away from oxidative phosphorylation toward glycolysis with disordered lipid metabolism (Wang et al., 2024).

## 3 Metabolic reprogramming in COPD

### 3.1 Mitochondrial dysfunction

Metabolically, COPD is strongly associated with chronic failures in mitochondrial quality control (MQC) (Li et al., 2024). Specifically, biogenesis is impaired by widespread mtDNA damage, ROS accumulation, and suppressed PGC-1α/ATP levels (Sm, 2020). Furthermore, compromised fusion–fission dynamics and insufficient PINK1–Parkin mitophagy degrade organelle integrity (Li et al., 2023).

These abnormalities directly compromise energy-demanding processes, including epithelial barrier maintenance and mucociliary clearance, and they favor cardiolipin oxidation, nucleoid destabilization, and leakage of mitochondrial DAMPs that activate NLRP3, promote IL-1β and IL-18 maturation, and fuel an energy deficit, oxidative stress, and inflammatory amplification cascade (Li et al., 2024; Lin et al., 2022). In parallel, downregulation of SIRT3 across COPD lung tissue and airway epithelium weakens antioxidant defenses and disturbs control of oxidative phosphorylation (Zhang et al., 2020).

Experimental restoration of SIRT3 activity or improvement of MQC has been shown to reduce mitochondrial stress, lower inflammatory burden, and partially restore high-energy epithelial functions (Ning et al., 2022). Persistent MQC failure, by contrast, prevents re-establishment of a mitochondrial state with low oxidative pressure, adequate ATP supply, and efficient mitophagy, thereby laying the metabolic foundation for subsequent glycolipid reprogramming and long-term disruption of nutrient-sensing pathways (Dobl et al., 2021).

### 3.2 Glycolytic and lipid metabolic reconfiguration

Inflammation, hypoxia, and oxidative stress rewire substrate use in COPD (Sun et al., 2020). Under low oxygen or energetic stress, alveolar macrophages and neighboring structural cells increasingly depend on glycolysis for rapid ATP generation (Woods et al., 2022). This shift is driven by HIF-1α stabilization and induction of glycolytic regulators such as PFKFB3 and PKM2 (Palsson-McDermott et al., 2015); nuclear PKM2 further promotes pro-inflammatory gene expression (Shirai et al., 2016). Accumulation of lactate and succinate alters pH and signaling, facilitating IL-1β activation and a Warburg-like macrophage phenotype that sustains inflammation but impairs phagocytosis (Sharma et al., 2020). Limiting glycolytic flux can mitigate fibrosis in chronic lung disease (Lu et al., 2024).

Lipid metabolism is likewise disturbed. Impaired fatty-acid oxidation and inefficient lipid-droplet turnover trigger lipotoxic stress (Hwang and Chung, 2021), while altered sphingolipid profiles and reduced ABCA1/ABCG1 expression hinder cholesterol efflux, fostering foam-cell formation and NLRP3 inflammasome activation (Groenen et al., 2021). Disordered membrane lipids compromise mitochondrial integrity, increase ROS production, and heighten susceptibility to cell death (Poulaki and Giannouli, 2022). Together, these changes lock macrophages into persistent inflammation and defective mucosal repair, perpetuating tissue injury even after external insults fade (Adolph et al., 2022; Bosch et al., 2020).

### 3.3 Abnormal nutrient-sensing pathways

In COPD, nutrient-sensing systems are chronically imbalanced. Sustained mTORC1 activation maintains anabolic S6K/4E-BP1 signaling while suppressing ULK1-dependent autophagy, trapping cells in oxidative and proteostatic stress (Liu and Sabatini, 2020). Inadequate AMPK activation further weakens bioenergetic adaptation and PGC-1α–driven mitochondrial biogenesis (Jäger et al., 2007). Declining NAD^+^ stores reduce SIRT1/3 activity, enhancing PGC-1α hyperacetylation, oxidative stress, and NF-κB signaling (Yang et al., 2009). Disrupted mTORC1–AMPK crosstalk plus a dampened NAD^+^–SIRT1 axis blocks autophagy and mitophagy and impairs mitochondrial restitution (Fang et al., 2014). By shifting the balance between glycolytic and lipid pathways, this disturbance stabilizes a state that favors glycolytic ATP production over oxidative phosphorylation while aggravating lipid derangements (Herzig and Shaw, 2018). Therapeutically, AMPK activation with partial mTOR inhibition alleviates epithelial stress and inflammation in models of tissue injury, and early studies suggest that NAD^+^ precursors may reduce airway inflammatory markers, although dosing and patient selection remain to be defined (Ma et al., 2023; Norheim et al., 2024).

## 4 Immune–metabolic crossroads and integration mechanisms

### 4.1 ROS–mTOR–AMPK axis

Persistent oxidative stress in COPD is not a uniform binary signal; rather, mtROS couples to nutrient-sensing in a cell-type- and stage-dependent manner. In alveolar macrophages and recruited monocytes, mtROS favors mTORC1 activation (e.g., p-S6) and AMPK inactivation, which suppresses ULK1-driven autophagy/mitophagy, promotes a sustained glycolytic, cytokine-high state, and reduces efferocytosis—thereby translating metabolic stress into innate immune aging. In parallel, in chronically stimulated CD8^+^ T and NK cells, mTOR–AMPK imbalance promotes terminal differentiation (CD28 loss/CD57 gain) and exhaustion-like dysfunction by limiting mitochondrial renewal and redox buffering (Ornatowski et al., 2020; Zhao et al., 2022; Liu et al., 2020; Liu et al., 2024; Ren et al., 2021). Notably, published data also indicate that epithelial mTOR activity can be compensatory early in cigarette-smoke injury, restraining epithelial cell death and airway inflammation; thus, mTOR inhibition is unlikely to be universally beneficial and may require cell-specific targeting and timing (Wang et al., 2018). Recent experimental evidence includes (i) human COPD airway/lung specimens and cigarette smoke extract (CSE)–exposed epithelial cultures showing altered mTOR/autophagy readouts and stress-induced cell death pathways, quantified by immunoblotting/IHC for p-mTOR/p-S6 and LC3B/p62 together with apoptosis/necroptosis markers (Wang et al., 2018); and (ii) chronic smoke-exposed mouse models in which AMPK activation with metformin attenuated airspace enlargement and inflammatory cytokines, with pathway engagement assessed by phospho-AMPK and downstream metabolic/mitochondrial assays in lung tissue and BAL cells (Cleveland and Cordoba-Lanus, 2021).

### 4.2 NAD^+^–SIRT1 pathway

Declining NAD^+^ availability provides a second integration node that links immunosenescence to mitochondrial quality control. In COPD, oxidative DNA damage and chronic inflammatory signaling can increase NAD^+^ consumption (e.g., via PARP activity) and shift NAD^+^ turnover, while myeloid and epithelial CD38 upregulation has been proposed as an additional sink, together lowering the NAD^+^ pool needed for SIRT1/SIRT3 deacetylase activity (Varveri et al., 2024; Zhang et al., 2022; Slama et al., 2024; Yarbro et al., 2020). Functionally, reduced SIRT1 in airway epithelium and macrophages weakens antioxidant programs, mitophagy/biogenesis (via PGC-1α deacetylation), and anti-inflammatory braking of NF-κB, thereby reinforcing SASP and inflammasome priming. Two complementary lines of recent evidence support this coupling: (i) a randomized clinical study of nicotinamide riboside in COPD demonstrated that boosting the NAD^+^ axis can reduce airway inflammatory readouts, providing direct human evidence supporting NAD^+^-targeting strategies (Norheim et al., 2024); and (ii) in cigarette-smoke COPD models, augmenting NAD(H) availability or activating sirtuin signaling reduced lung inflammation and oxidative injury, with mechanistic endpoints measured by ELISA for cytokines, Western blot for SIRT1/NF-κB acetylation status, and mitochondrial stress markers in lung tissue and airway cells (Slama et al., 2024).

### 4.3 NF-κB–SASP feedback loop

NF-κB-driven SASP is a contextual amplifier that connects injured structural cells to immune-cell metabolic reprogramming. In COPD airways, senescent epithelial cells, fibroblasts, and macrophages can produce SASP mediators (IL-1 family members, IL-6, CXCL8, TNF-α), which recruit and polarize myeloid cells and reinforce HIF-1α–linked glycolytic programs, while also sustaining antigenic/inflammatory pressure that promotes exhaustion-like lymphocyte phenotypes (Sm, 2020; Barnes et al., 2019; Alabdullah and Hadji-Ashrafy, 2022; Bruzzaniti et al., 2019; Kotlyarov, 2025). However, inconsistency across studies (e.g., which SASP factors dominate) likely reflects differences in sampling compartment (sputum vs. BAL vs. tissue), disease state (stable vs. exacerbation), and analytical platform; harmonized panels and spatial readouts are therefore needed to map SASP microenvironments rather than relying on single-cytokine conclusions. Recent experimental evidence includes (i) *ex vivo* air–liquid interface differentiation of primary bronchial epithelial cells from COPD donors showing persistent p16/p21 senescence with SASP secretion, and pharmacologic senolysis (dasatinib+quercetin) reducing inflammatory outputs and improving epithelial phenotype (Baker et al., 2025); and (ii) *in vitro* cigarette smoke extract (CSE)-stimulated human bronchial epithelial cells (16HBE) in which perturbation of the XIST/miR-200c-3p/EGR3 axis modulated apoptosis and inflammatory cytokine readouts, quantified by RT-qPCR/Western blot and cytokine assays, linking NF-κB–adjacent transcriptional control to epithelial senescence-like programs (Chen et al., 2021).

### 4.4 Mitochondrial DAMPs–NLRP3 pathway

Mitochondrial DAMPs provide a danger-signal axis that converts mitochondrial quality-control failure into sustained innate cytokine release. Oxidized mtDNA, cardiolipin, and extracellular ATP released from stressed epithelium and dying immune cells can be sensed by primed macrophages and neutrophils, promoting NLRP3–caspase-1 activation, IL-1β/IL-18 maturation, and (in some settings) pyroptotic cell death that further increases DAMP burden (Swanson et al., 2019; Wu et al., 2019; Iyer et al., 2013; Chen et al., 2023). Cigarette smoke–associated gasdermin D activation provides mechanistic support for pyroptosis-like chronic pulmonary inflammation in COPD (Quesniaux et al., 2022). Importantly, the magnitude of NLRP3 activation appears context dependent: it is often more pronounced during exacerbation-like conditions or combined pollutant exposures, which may explain discrepant findings in stable-disease cohorts. Two recent experimental lines illustrate this: (i) a translational study integrating COPD patient data with mouse and human bronchial epithelial cell experiments showed that combined cigarette smoke and PM2.5 exposure aggravates lung injury via the NLRP3/caspase-1 pathway, using patient correlation analyses, murine emphysema readouts, and mechanistic assays for caspase-1/IL-1β activation (Chung et al., 2024); and (ii) a case–control human study of stable COPD measuring plasma IL-1β (Luminex) together with IL1B/NLRP3/CASP1 gene expression (TaqMan qPCR) in peripheral blood reported coordinated upregulation of the systemic NLRP3 axis, supporting that basal inflammasome tone may vary by cohort and sampling compartment even outside exacerbations (Markelić et al., 2022).

### 4.5 Integrated model: the immune–metabolic positive feedback loop in COPD

Persistent oxidative stress, disturbed energy sensing, and defective autophagy favor retention of damaged mitochondria that continuously leak danger signals (Rivas et al., 2022). A weakened NAD^+^–SIRT1 axis reduces mitochondrial renewal and antioxidant defenses, whereas the NF-κB–SASP network and mitochondrial DAMPs–NLRP3 cascade expand low-grade inflammation in time and space (Bateman et al., 2023; Ramos Jesus et al., 2024). An immune–metabolic positive feedback loop thereby couples immune senescence to metabolic reprogramming, allowing airway inflammation and tissue injury to persist after external triggers subside and pushing COPD toward structural aging and irreversible functional decline (Easter et al., 2020).

## 5 Potential biomarkers

Because no single marker captures the coupled immune–metabolic loop, biomarker development should be organized around (i) immune aging/exhaustion, (ii) inflammaging/SASP burden, (iii) mitochondrial–metabolic stress, and (iv) integrative multi-marker panels that reflect node combinations rather than isolated pathways.

For immune aging/exhaustion, peripheral blood and airway T-cell phenotypes (CD28 loss with CD57/KLRG1 gain, together with inhibitory receptors such as PD-1 when supported by functional impairment) plus Th17/Treg balance readouts provide a mechanistically grounded axis linked to severity and exacerbation risk (Xue et al., 2024; Fernandes et al., 2022). In parallel, myeloid-function markers—such as impaired phagocytosis/efferocytosis signatures in alveolar macrophages captured by high-dimensional flow cytometry or single-cell RNA sequencing—can complement lymphocyte-centric indices and help anchor biomarkers to dominant cellular drivers of the loop (Bassler et al., 2022).

For inflammaging, multiplexed measurement of SASP-associated cytokines (e.g., IL-6, IL-8, TNF-α, IL-1β/IL-18) in sputum, BAL, or exhaled breath condensate can quantify the inflammatory set point, but should ideally be interpreted alongside senescence markers (p16/p21) and spatial localization to distinguish true senescence niches from acute inflammation (Baker et al., 2025; Kumar et al., 2014). For mitochondrial–metabolic stress, circulating NAD^+^/NADH-related metrics and SIRT1 activity report energy-sensing capacity, while cell-free mtDNA and leukocyte mtDNA damage/copy-number changes track mitochondrial injury and have been associated with COPD progression (Zhang et al., 2021). Accordingly, a defensible translational strategy is to use a composite immunometabolic panel that combines immune-aging signatures, SASP mediators, mitochondrial injury markers, and inflammasome activity, and tests incremental predictive value beyond eosinophils or BODE using pre-specified feature reduction (PCA/PLS), penalized modeling, and external validation cohorts (Markelić et al., 2022). Importantly, early human intervention data (e.g., NAD^+^ boosting with nicotinamide riboside) can be leveraged to qualify responsive biomarkers and define on-target signatures for stratified trials (Norheim et al., 2024).

## 6 Future challenges and research prospects

Moving the immune–metabolic vicious-cycle model into routine clinical use is still limited by a substantial gap between experimental systems and real-world COPD. Much of the available evidence comes from short-term or single–time point studies and from young-animal models that do not capture the cumulative impact of aging, multimorbidity, and long-term environmental exposure (Easter et al., 2020). Future research should therefore place aging at the center of study design and use longitudinal cohorts covering a wide range of ages and phenotypes (Kakkera et al., 2023).

Integration of single-cell and spatial multi-omics with metabolic-flux profiling and high-dimensional immunophenotyping will be crucial for dynamically tracking mitochondrial DNA damage, NLRP3 activity, immune exhaustion signatures, and SASP trajectories (Rojas-Quintero et al., 2024). Carefully designed perturbation studies are also needed to clarify the causal order and maintenance of these interconnected pathological processes (Auffray et al., 2010).

Important methodological gaps remain. Cellular maps are still incomplete for pulmonary B-cell niches, tertiary lymphoid structures, and senescent NK and MAIT-cell subsets, as well as for their interactions with epithelial and fibroblast lineages. Mechanistic understanding of lung–skeletal muscle crosstalk in frailty and sarcopenia is similarly limited, even though this relationship likely contributes substantially to systemic functional decline (Madissoon et al., 2023).

A growing set of low-intensity, druggable interventions is emerging. Here, low-intensity multi-target approaches denote partial, network-level modulation—either via low-dose combinations or multi-target single agents—aimed at tuning (not fully blocking) coupled nodes such as SASP, mitophagy, NAD^+^–SIRT1, AMPK–mTOR, and NLRP3 to reduce adverse effects and escape single-pathway redundancy (Baker et al., 2025; Ryter et al., 2018). Candidate approaches include senolytic/senomorphic regimens to blunt SASP, agents that enhance mitophagy or restore NAD^+^–SIRT1 signaling, AMPK activation with modest mTOR restraint, selective NLRP3 inhibition, and microbiota-targeted modulation along the lung–gut axis (Qu et al., 2022). Key risks include unpredictable off-target effects, drug–drug interactions, impaired host defense, and regulatory complexity; therefore, dose-finding, safety monitoring, and biomarker-guided stopping rules are essential. Given the above, heterogeneity and endotype structure should be treated as a cross-cutting design variable rather than an afterthought, and we discuss mechanism-driven stratification below.

Early trials of nicotinamide riboside have reported reductions in airway inflammatory markers, and observational studies suggest that metformin use may be associated with lower exacerbation and hospitalization risk, but prospective trials are needed; evidence for NLRP3 inhibition remains mainly preclinical (Norheim et al., 2024). To support translation, studies should pair therapies with composite immunometabolic biomarkers as companion diagnostics and adopt mechanistically guided, stratified enrollment with adaptive dosing and real-world monitoring (Leung et al., 2019).

### 6.1 Heterogeneity and endotypes: structuring immunometabolic stratification

COPD heterogeneity (e.g., eosinophilic vs. neutrophilic inflammation, emphysema- vs. bronchitis-predominant disease, and frequent-exacerbator phenotypes) and comorbidities (cardiovascular disease, obesity, metabolic syndrome) are expected to reshape the proposed immune–metabolic circuitry by changing which cell types dominate and which node becomes rate-limiting. Systemic metaflammation can prime lung myeloid and epithelial cells toward higher basal NF-κB/NLRP3 tone, thereby lowering the activation threshold of the reinforcing loop. For instance, obesity-related adipose inflammation can amplify systemic IL-6/TNF signaling and lipid stress, potentially increasing pulmonary macrophage foam-cell programs, mtROS, and NLRP3 tone, Whereas microbial colonization and gut–lung axis perturbations may preferentially sustain antigenic stimulation and exhaustion-like lymphocyte dysfunction (Qu et al., 2022). Conversely, eosinophilic/Type-2–skewed disease may reflect alternative inflammatory wiring in which parts of the inflammasome–SASP axis are less dominant. Recent single-cell profiling of the alveolar compartment in COPD has shown expansion of distinct monocyte–macrophage states with altered functional programs, supporting the idea that immunometabolic set points differ across patient subsets and sampling compartments (Bassler et al., 2022). Spatial multi-omics further indicates that immune niches (e.g., B-cell–rich or tertiary-lymphoid–like regions) are unevenly distributed in COPD lungs and may couple to different remodeling trajectories, implying that the feedback loop may be locally strong in some microenvironments but weak in others (Rojas-Quintero et al., 2024). A practical next step is mechanism-driven classification that assigns patients to dominant node patterns (e.g., SASP-high, NLRP3-high/mtDNA-high, NAD^+^–SIRT1-low, glycolysis/lipid-stress–high) and tests whether these endotypes better predict outcomes and treatment response than clinical labels alone, using standardized sampling, longitudinal design, and pre-registered analytic pipelines to reduce between-study inconsistency.

In parallel, further development of humanized aging models, multi-organ interaction platforms, standardized immunometabolic biomarker systems, and clinically applicable staging and risk-stratification frameworks will be essential for moving from mechanistic insight to precision intervention in COPD (Wu et al., 2025).

## 7 Conclusion

We propose COPD as an age-accelerated disorder maintained by a self-reinforcing immune–metabolic feedback loop centered on mitochondrial dysfunction. Across four cross-links—mtROS-driven mTOR–AMPK imbalance, NAD^+^–SIRT1 depletion, NF-κB–SASP signaling, and mitochondrial DAMP–NLRP3 activation—immune senescence/exhaustion and metabolic reprogramming reciprocally amplify chronic low-grade inflammation and defective repair. This framework connects biomarkers (immune-aging signatures, SASP mediators, mitochondrial injury and inflammasome activity, nutrient-sensing readouts) to a translational strategy using composite panels for risk prediction, endotyping, and response-guided multi-node interventions.

Because COPD is heterogeneous, parallel drivers (epithelial fragility, protease–antiprotease imbalance, extracellular matrix remodeling, and microbiome disruption) may dominate in subgroups. Integrating immunometabolic endotyping with imaging and clinical indices may enable mechanism-informed stratification and more rational trial design for precision interventions that target aging biology.
